# *A* solar energy desalination analysis tool, *sedat*, with data and models for selecting technologies and regions

**DOI:** 10.1038/s41597-022-01331-4

**Published:** 2022-05-20

**Authors:** Vasilis Fthenakis, Gregory Yetman, Zhuoran Zhang, John Squires, Adam A. Atia, Diego-César Alarcón-Padilla, Patricia Palenzuela, Vikas Vicraman, Guillermo Zaragoza

**Affiliations:** 1 Center for Life Cycle Analysis, School of Engineering and Applied Science, Columbia University, 500 W, 120th street New York, New York, NY 10027 USA; 2 CIESIN - Center for International Earth Science Information Network, Columbia University, 61 Route 9 W, Palisades, NY 10964 USA; 3 CIEMAT - Plataforma Solar de Almería. Ctra. Senés s/n, 04200 Tabernas, Almería, Spain

**Keywords:** Energy modelling, Environmental impact, Solar thermal energy

## Abstract

There is interest for desalination technologies powered by solar energy as arid areas are typically bestowed with good solar potential. In response to a US DOE call for solar desalination analysis tools, we developed an open-source solar energy desalination analysis tool, *sedat*, for techno-economical evaluation of desalination technologies and selection of regions with the highest potential for using solar energy to power desalination plants. It is expected that this software will simplify the planning, design, and valuation of solar desalination systems in the U.S. and worldwide. *Sedat* uses Dash for integrating various layers of large volumes of GIS data with Python-based models of solar energy generation and desalination technologies. It derives time-series of energy generation and water production, with details of plant performance and suggestions for improving the solar-desalination coupling. This paper summarizes the various phases of the tool’s development, presents example results showing the potential, under multiple objectives, of solar desalination in parts of the U.S. southwest, and discusses method details that would be useful for future model development.

## Introduction

Producing fresh water via desalination is essential for arid, water-scarce regions, but it is expensive and energy-intensive. The cost of energy is a significant contributor to this high cost, and the use of fossil fuels that currently power desalination plants causes emissions of greenhouse gases and other hazardous pollutants. However, the recent cost reductions and technological advances of solar energy systems create opportunities for implementing low-cost and emission-free desalination technologies.

This paper discusses the development of *sedat*, a user-friendly software for enabling a comparative evaluation of solar desalination technology options and using geospatial data layers to identify regions of high-potential for solar thermal desalination. *Sedat* uses geospatial analysis in combination with an energy and desalination technology modeling framework that describes current and emerging desalination processes on industrial scales. Its use enables: (i) streamlined identification of locations where solar thermal desalination can be most competitive and (ii) system-level simulation & optimization of solar energy and desalination system integration. It is expected that this software will simplify the planning, design, and valuation of solar desalination systems in the U.S. and worldwide.

*Sedat* is an open-source, Python-based tool, that integrates various layers of data describing solar and saline water resources, water and energy infrastructure, applicable regulations, costs and competitive prices. Most of these data, except for the solar irradiation and United States Geological Survey (USGS) water resources, were not available in a single database. Thus, we integrated a suite of geospatial data sources and techno-economic input parameters, shown in Table 1, for simulating integrated solar energy technology systems and desalination technologies in one graphical user interface (GUI) that can be used and efficiently processed in desktop and laptop computers.

**Table 1:** Primary data layers integrated into *sedat*.

Parameters	Data layers	Sources
Water Demand	Population density & projections	Hauer^19,28^
	Canals and aqueducts	USGS^19^
	Roads as water network proxy	U.S. Census Bureau^20^
Alternative Water Sources	Brackish water sources: depths, total dissolvable solids (TDS)	USGS^32^
	Oil & Gas Produce water TDS	USGS National Produced Waters Data^15^
	Desalination plants in the U.S.	Global Water Intelligence (GWI)^17^, Texas Water Development Board^33^
Solar Resources	Solar Irradiance: GHI, DNI	NSRDB^10^, PVGIS^11^
Heat Sources	Power plant	EIA^34^
	Water temperature	USGS (brackish)^32^
Land Use	Topography	MapBox^35^
	Restricted and sensitive areas	USGS^36^
	Agricultural saline water drainage regions	CA-Dept. Water Resources^37^
Cost Data	Municipality water prices	IBNET^23^
	Cost data for desalination systems	Multiple literature sources^38–42^
	Utility electricity rate structures	OpenEI^43^
	Average fuel prices	U.S. Energy Information Administration API^44^
Planning, Regulatory	Desalination & water treatment regulatory and permitting requirements	State Water Resource Control Boards^22–27^

Siting a solar desalination facility requires information on a variety of inputs related to resources (solar inputs, water sources), markets (energy and water prices), and legal frameworks (e.g., permitting requirements and land use restrictions). Selecting and appropriating solar desalination technology requires models based on both location-specific characteristics and non-spatial input values. We have assembled a collection of integrated spatial data inputs and implemented a map-based data exploration interface with the ability to choose a location, solar technology model, and cost model to produce a report and charts of system inputs and outputs to evaluate and compare different options for technology and site selection.

For example, for solar desalination of inland brackish water, the USGS data are used to seed the database with known brackish water resources, and existing water infrastructure (e.g., national level water networks like National Hydrography Dataset (NHD)^1^, as well as state level networks such as California Central Valley Project (CVP) and State Water Project (SWP), represented by aqueducts). The choice of technology can be identified based on the quality and quantity of water, the brine management requirements, the solar resource and the electricity & fuel prices in the region.

The software also allows the user to enter data and parametric inputs on geospatial, economic, and technical variables. For example, a user may provide an alternative source (perhaps from more detailed, proprietary, or up-to-date data) in place of one or more of the input data that are available within the software. Such an approach contributes to product flexibility and to the potential of creating a data hub for future use. Alternative data sources can be provided through the GUI manually or by updating the data files available within the application.

**Fig. 1:**
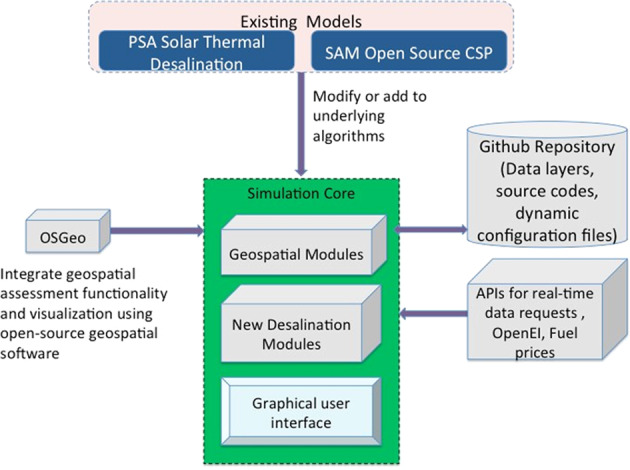
Basic framework of *sedat*.

A web interface was developed for quick visualization of geospatial data and performing simple techno-economic calculations without requiring software installation, while an integrated GIS and Python application was developed for more detailed geospatial and techno-economic analyses. The desktop application includes comprehensive techno-economic models of desalination technologies developed during this project that facilitate the most effective use of solar thermal energy. This model development leverages existing tools, like the concentrated solar power (CSP) models in National Renewable Energy Laboratory’s (NREL) SAM open-source^2^, and solar thermal energy and desalination models available from researchers at CIEMAT-Plataforma de Almeria (PSA)^3^. Figure 1 shows a high-level framework of the tool.

The software uses a modular architecture in its design making it flexible for expansion. The techno-economic modules deliver an analytical workflow for planning and designing solar thermal desalination systems in optimal locations via a user-friendly GUI. This interface provides default value inputs for technical design as well as capital and operating cost parameters to allow for comparative analyses between different solar thermal desalination technologies as well as other competing desalination technologies. The software quantifies the performance of user-selected desalination systems based on regional specifications and cost parameters, while listing the underlying assumptions. The user can change the assumptions and select another system of interest for evaluation. The technology suggestions are based mainly on the saline water total dissolvable solid (TDS) concentration, levelized cost of product water (LCOW), product purity, and target brine concentration.

The desalination techno-economic models include: Low-temperature multi-effect distillation (LT-MED), multi-effect distillation with thermal vapor compression (MED-TVC), multi-effect distillation with absorption heat pumps (MED-ABS); vacuum air gap membrane distillation (VAGMD) in continuous and batch operation modes, reverse osmosis (RO) with multiple passes, osmotically assisted reverse osmosis (OARO), forward osmosis (FO), and RO-VAGMD, and RO-FO hybridizations.

**Fig. 2:**
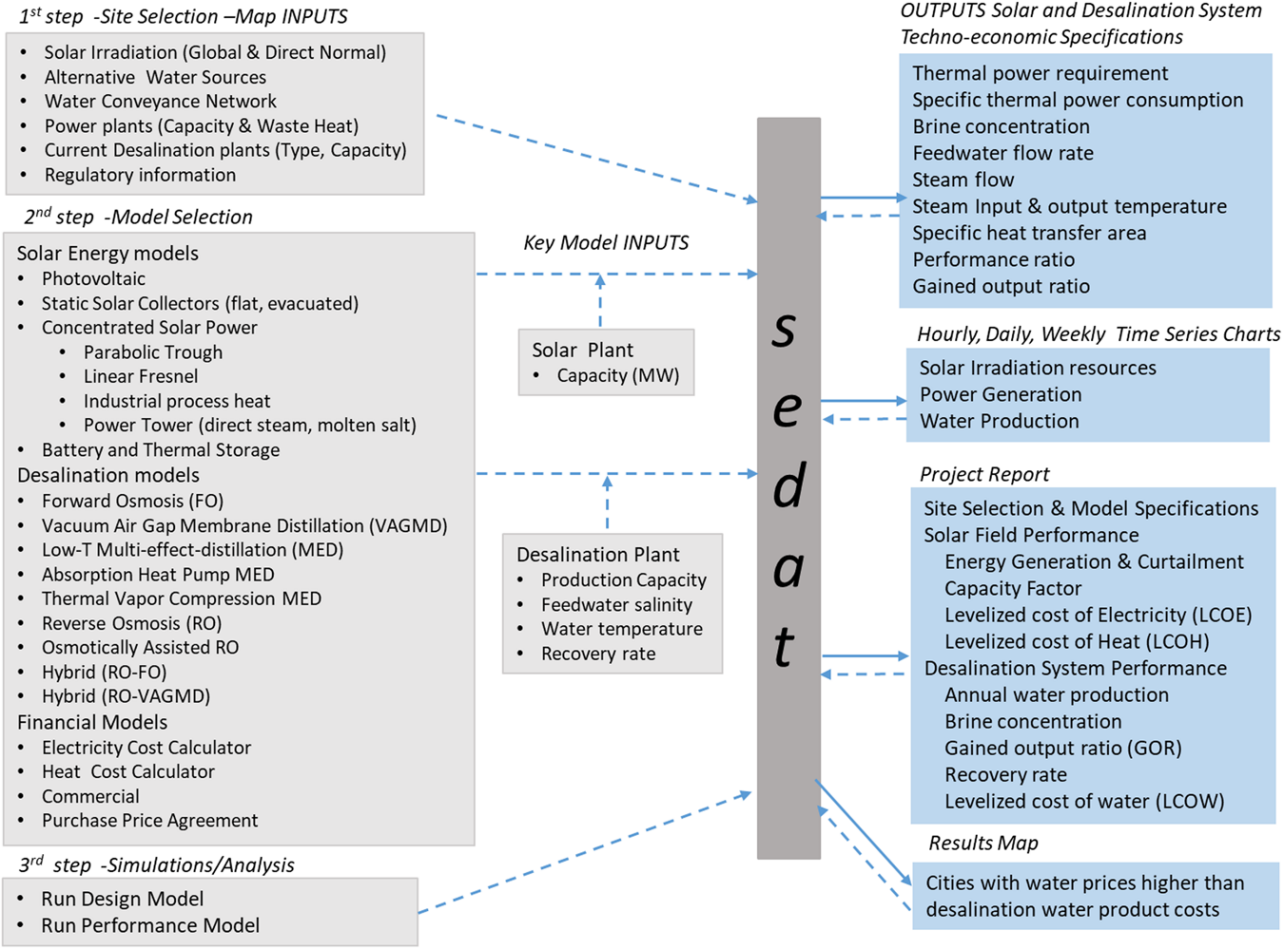
Inputs and outputs of *sedat* and sequence of generating results for analysis of solar desalination technologies in considered regions.

The user interface goes over the subsequent steps of selecting a site of interest, selecting the technical and financial technology models, running desalination plant design and performance simulation, and showing results in terms of plant specifications and performance time-series. Figure 2 gives an overview of this architecture. The left columns list the inputs and the right one lists the outputs.

Software modules based on open geographic software libraries from OSGeo^4^ and other sources assemble, extract, transform and pass the geospatial data to techno-economic solar generation and desalination components. A logic was implemented to dynamically collect model input parameters from locations in the GUI. Once a user selects a location, the associated values for the variables needed in the solar and the desalination models are displayed to the user and stored in JavaScript Object Notation (JSON) file for using as model inputs after the model selection. This process can be repeated iteratively using different locations; once a user is satisfied with a location selection, they can select models of solar generation, desalination and financials. Variables that were derived from the geographic maps and used as input into the models are displayed together with the model results in a summary report and results map.

## Results

The open-source software libraries used in the application support the GUI and analysis functions. Both Dash-Plotly with Dash-Leaflet are used for visualization (i.e., maps, table display) and user input data. Using only the Dash-Plotly map application required that all data be stored in computer memory. This degraded performance at load time when starting the map application and when different map themes (e.g., legal restrictions, waste heat) were chosen by the end user. Also, the selection of a new site by the user required a reload, making this process slow. To solve this issue, a new map framework was implemented for Dash, based on a web map framework known as Leaflet (https://pypi.org/project/dash-leaflet/) This allows layers to be retrieved from Mapbox and loaded locally. The new framework enables dramatic improvements in load time and greatly improves performance when a user selects a site, with near-instantaneous response from the application when panning and zooming.

Using this framework, we implemented a prototype that enabled the following: Splitting data for faster querying, ability to display and allow users to edit input values, ability to call external Python modules from the framework, simplifying the interactions with technologies and protocols, and displaying an interactive map. The framework allows adding new solar and desalination models, serving the objective of building a modular, expandable tool for solar desalination analyses.

### Site selection

**Fig. 3:**
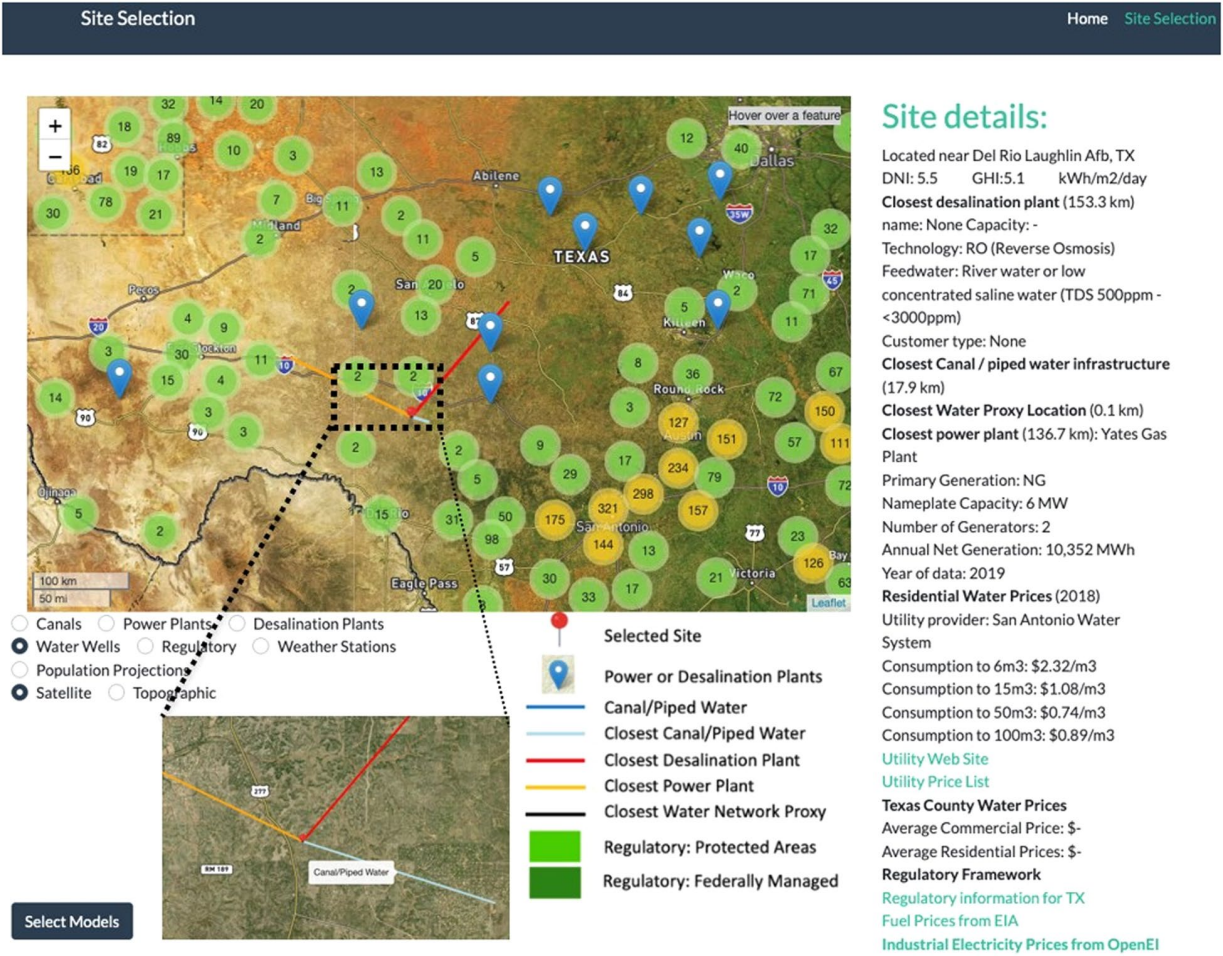
Example Site Selection and associated display information.

An example of a site selection result is shown in Fig. 3. The theme dropdown in the left of the figure can be used to view various geographic data sets, such us solar resources, water prices, and brackish water resources. Spatial objects are also added to the map where appropriate; for instance, while looking at existing power plants, a line between the selected site and the closest power plant and the closest desalination plant is shown. In different themes, a different line may be shown, such as a line to link the selected site to the closest water transportation infrastructure in *sedat’s* GIS database.

If a user has already selected a point, the location chosen is preserved with the change of theme. In any of the implemented themes, the user can choose a new location and proceed to model selection. By zooming on a location the user can see all the terrain infrastructure details available from satellite and topographical maps.

### Solar generation model development

CSP and PV models associated with corresponding financial models were integrated from SAM so that the user has full access to all the inputs and outputs in SAM -a widely used solar simulation software. Static collector models developed by PSA were translated into Python and were normalized to share the same data as the SAM solar models. The weather information is linked to the user’s location selection on the map. *Sedat* includes major outputs that were not included in the original models, such as waste heat generation which is depicted by time-series charts and summary reports, while details can be exported through Excel files.

### Desalination model development

Desalination techno-economic models were unified in terms of code structure and data frame. Technologies were described by individual design and simulation models. The former provides the physical design (e.g., specific heat exchange area) and the performance of system at the design point within a few seconds, and it allows the user to adjust the system parameters accordingly. A simulation model reads the hourly generation from the solar model and executes cost simulations to provide a complete analysis of the annual performance of the system.

### Integration of solar and desalination models

As shown in Fig. 2, the user can select from nine solar energy generation, nine desalination and four cost models. In this section we present just four examples of such selection and integration of models in *sedat* simulations: a) flat-plate solar collectors with membrane distillation (MD), b) flat-plate solar collectors with low temperature multi-effect distillation (LT-MED), c) linear Fresnel CSP with LT-MED and d) parabolic trough CSP and thermal storage with MED.

#### Solar flat-plate collectors and MD

**Fig. 4:**
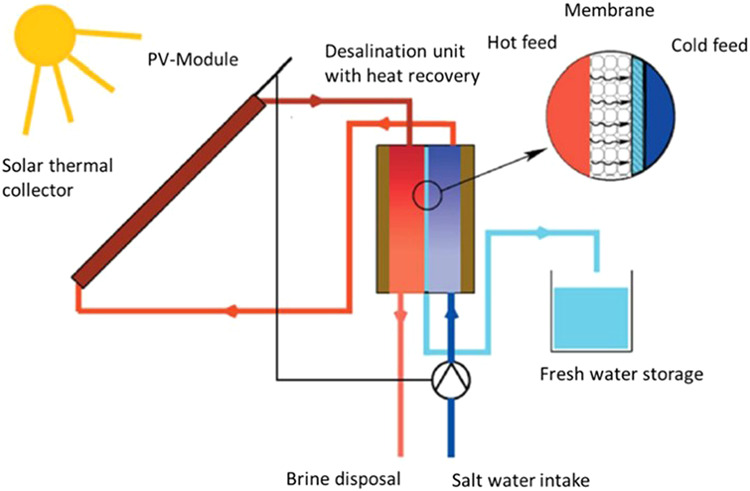
Schematic of flat plate collector and MD system^49^.

**Fig. 5:**
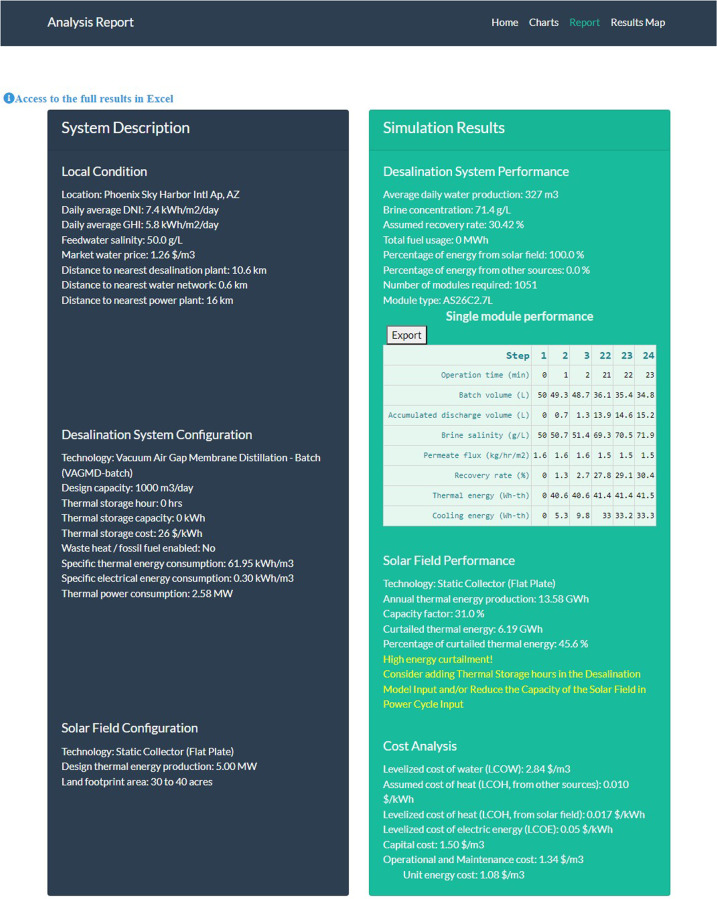
Example simulation report showing local conditions, desalination and solar field configurations, and techno-economic performance of the batch VAGMD system with flat plate collectors (w/o TES).

**Fig. 6:**
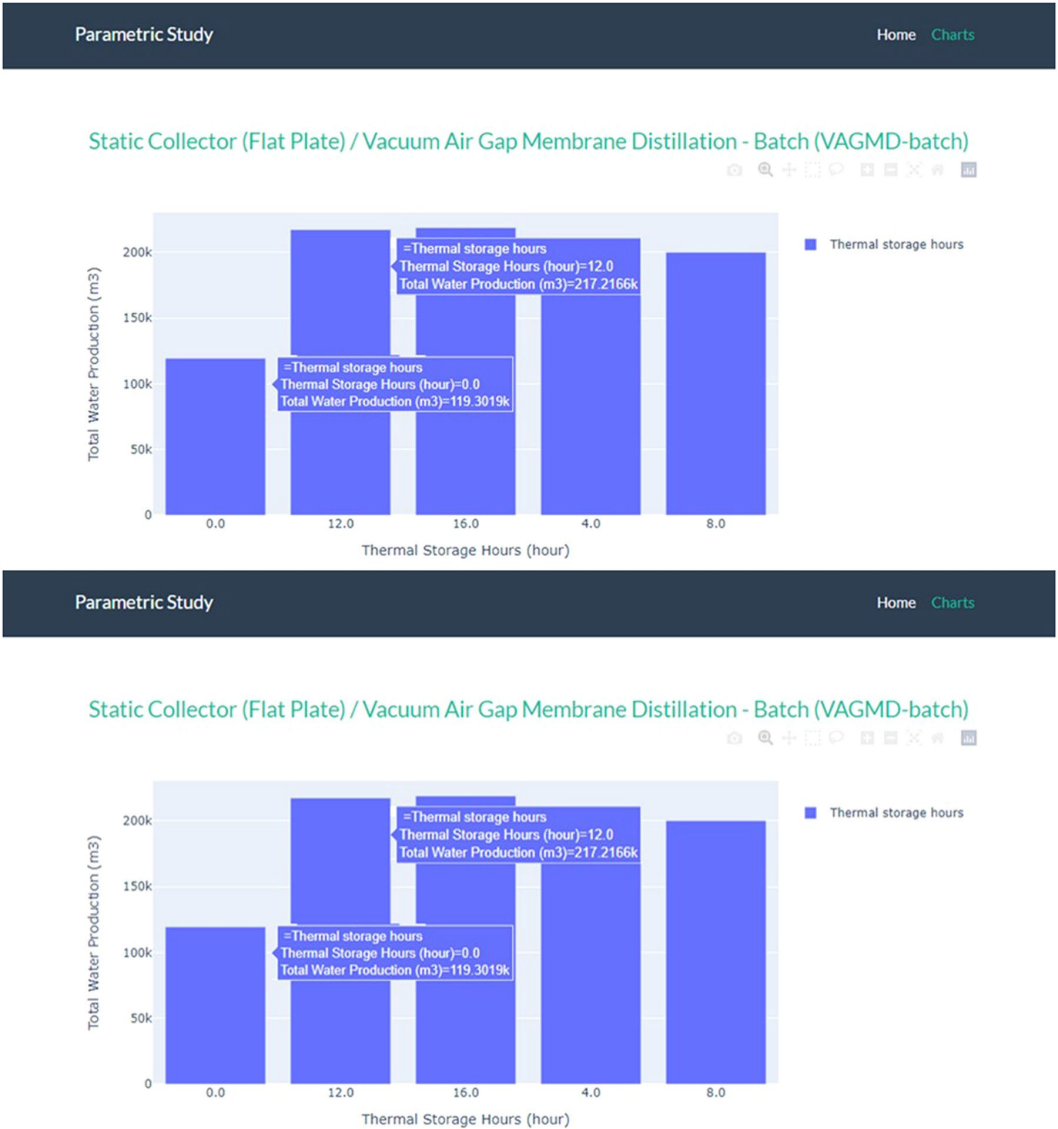
Example parametric analysis result on the size of TES for flat plate collectors with a batch VAGMD plant in *sedat*.

**Fig. 7:**
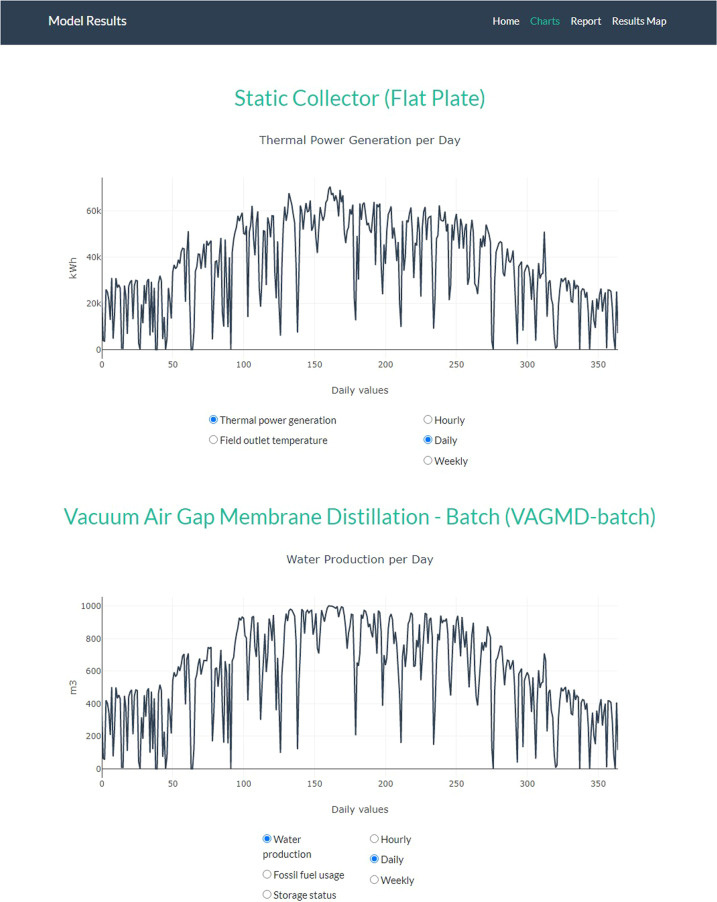
Example time-series of outputs for flat plate collectors with a batch VAGMD plant (with a 12-hr TES) visualized in *sedat*.

Among current MD designs, vacuum-enhanced air-gap (VAGMD) offers the lowest specific thermal energy consumption (STEC) and when operating in batch mode, it also offers high recovery rates. Empirical models were developed based on pilot systems designed by Zaragoza and co-workers^5^ for both one-pass and batch VAGMD configurations. These were integrated with the flat-plate collector model described earlier. A schematic of this system is shown in Fig. 4. Figure 5 shows an example of the associated Results Report. Figure 6 shows a parametric analysis on the integration of thermal energy storage (TES) at different sizes, and Fig. 7 shows time-series plots of daily thermal power generation and water production for the system.

In the above example, a message warning about high energy curtailment was displayed as such curtailment was above a threshold of 20%. In most cases the user can follow the instructions to involve the TES or reduce the size of solar field. The message also offers suggestions for resolving the issue; namely either reducing the size of the solar system or adding energy storage to it. Subsequently, the parametric analysis option in sedat was used to examine various thermal energy storage sizes.

As shown in Fig. 6, parametric analysis of the considered system indicated that 12 hr. of thermal storage is the most cost-effective TES option for the considered system and location. At that level the TES uses all thermal energy from a 5 MW_e_ solar collector and it produces 140% more water at 15% lower LCOW compared to the reference case without a TES. Sedat produces hourly, daily and weekly time-series of the system performance. Figure 7 shows the daily energy generation and water production of the CSP + 12 hr TES.

#### Flat plate collectors and LT-MED

**Fig. 8:**
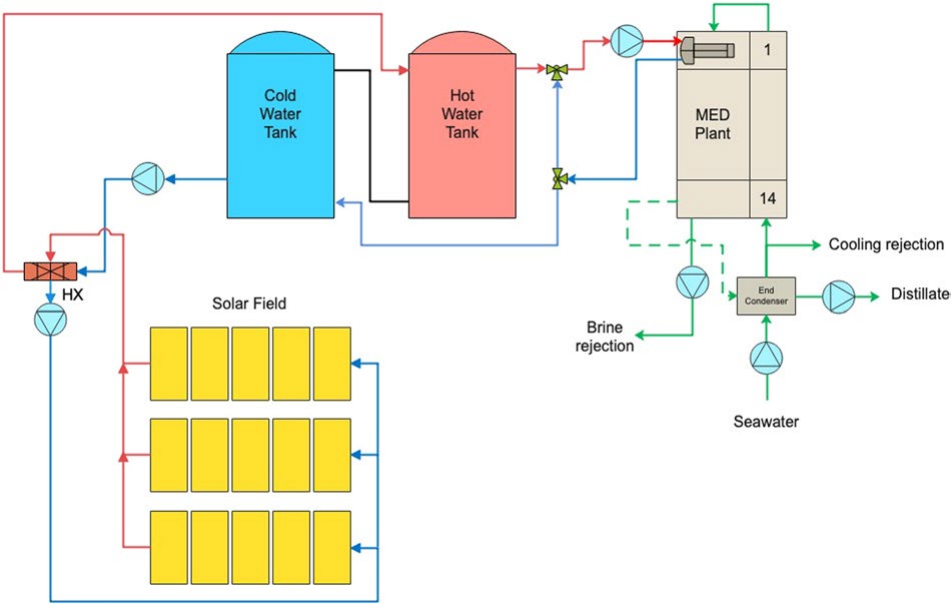
Schematic of the solar filed composed of flat plate collectors and LT-MED plant at PSA.

This model is based on the performance of a solar thermal LT-MED desalination pilot plant operating in Plataforma Solar de Almeria (PSA), Spain^6^ (Fig. 8). The MED system is comprised of 14 effects and 13 preheaters using solar energy from AQUASOL-II, a solar field with 60 flat-plate collectors. The overall thermal energy demand for the MED system is 190 kW at 70 °C^7^.

Preliminary simulations of this integrated system showed that the LT-MED model needed to be modified. The original model was intended for guiding the design of a test pilot plant, estimating the optimal heat exchange area in each effect. The LT-MED model was transformed from a design to a simulation tool, where heat exchange areas in each effect are constant, and the multiple-effect temperatures were calculated according to the input conditions (e.g., steam temperature at the first effect, temperature at the final condenser, and feed water flow).

#### Linear Fresnel Direct Steam CSP and MED

**Fig. 9:**
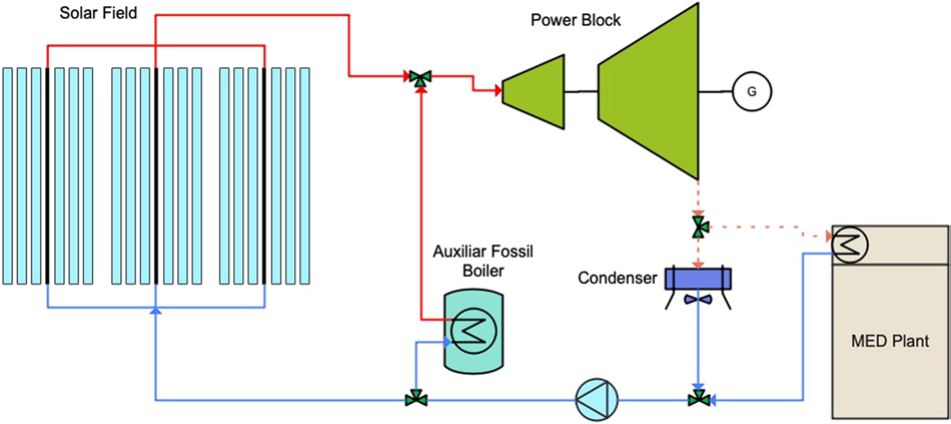
MED unit integrated into a Linear Fresnel Direct Steam CSP plant.

Another integration of CSP and thermal desalination includes a Linear Fresnel with Direct Steam Generation (LF-DSG) CSP component and the aforementioned LT-MED model^8^ (Fig. 9).

**Fig. 10:**
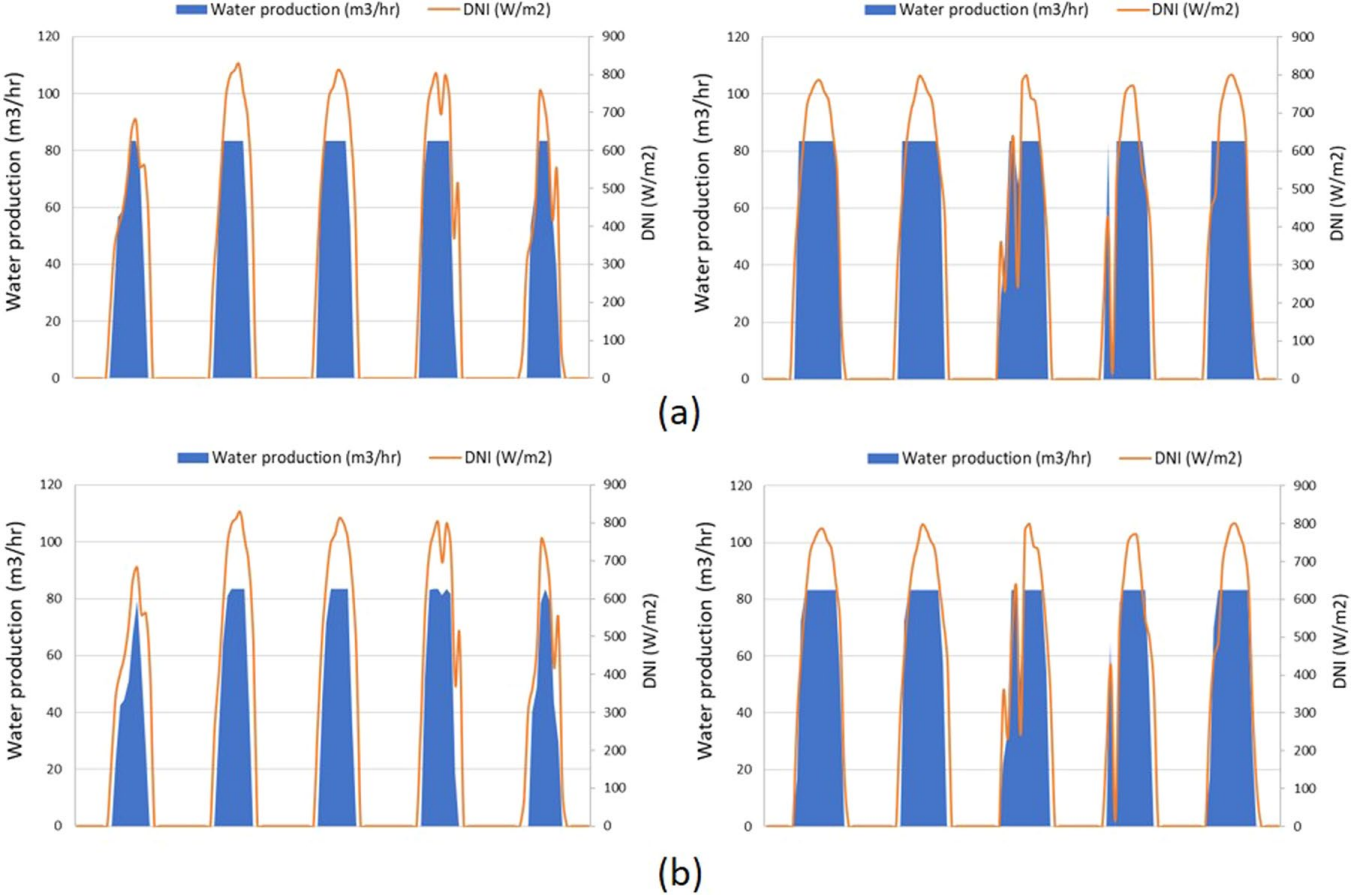
Modeled water production in Abu Dhabi during January 24–28 (two images on the left), and during May 21–25 (right images) with different sizes of LF-DSG solar fields; (**a**) 2.5 MW_e_ LF-DSG with 2000 m^3^/day LT-MED; (**b**) 1.8 MW_e_ LF-DSG with 2000 m^3^/day LT-MED.

Figure 10a shows the hourly simulation of 2.5 MW_e_ LF-DSJ powering an LT-MED plant (2,000 m^3^/day) fed with 60 g/L saline water. The simulation gave a “High energy curtailment warning” and a smaller solar field (i.e., 1.8 MW_e_) is selected to reduce the energy curtailment as instructed in the warning message; the time-series of this simulation is shown in Fig. 10b.

By reducing the size of the solar field from 2.5 MW_e_ to 1.8 MW_e_, the thermal energy curtailment drops from 31.7% to 12.2%, at the sacrifice of ~5.8% water production through a year. As shown in Fig. 10, the water productions are similar in May as there is excess energy generated, and the desalination system operates at full capacity during sunny days. In cloudy days when irradiation is insufficient, less waste energy is produced from the 1.8 MW_e_ CSP system and correspondingly the water production is less.

#### Parabolic trough CSP and MED

A model of an integrated system comprising parabolic trough (PT) CSP and an MED pilot plant was developed based on the specifications of PT and MED pilot plants at PSA, Almeria, Spain^9^. The 14-effect LT-MED system powered by a 50 MW_e_ PT-CSP system, produces water at 42,927 m^3^/day. The MED model was integrated with the hourly simulations from SAM to describe the dynamic performance of an electricity and water cogeneration desalination plant. In this system, the thermal storage supplies the required thermal power for power generation and cogeneration during night time. The turbine exhaust steam temperature is not provided by SAM; thus, it is calculated as the sum of the dry bulb temperature and the initial temperature difference between steam and ambient temperature. The calculated turbine exhaust steam temperature becomes an input to the steam temperature at the inlet of the tube bundle of the MED plant’s first effect.

**Fig. 11:**
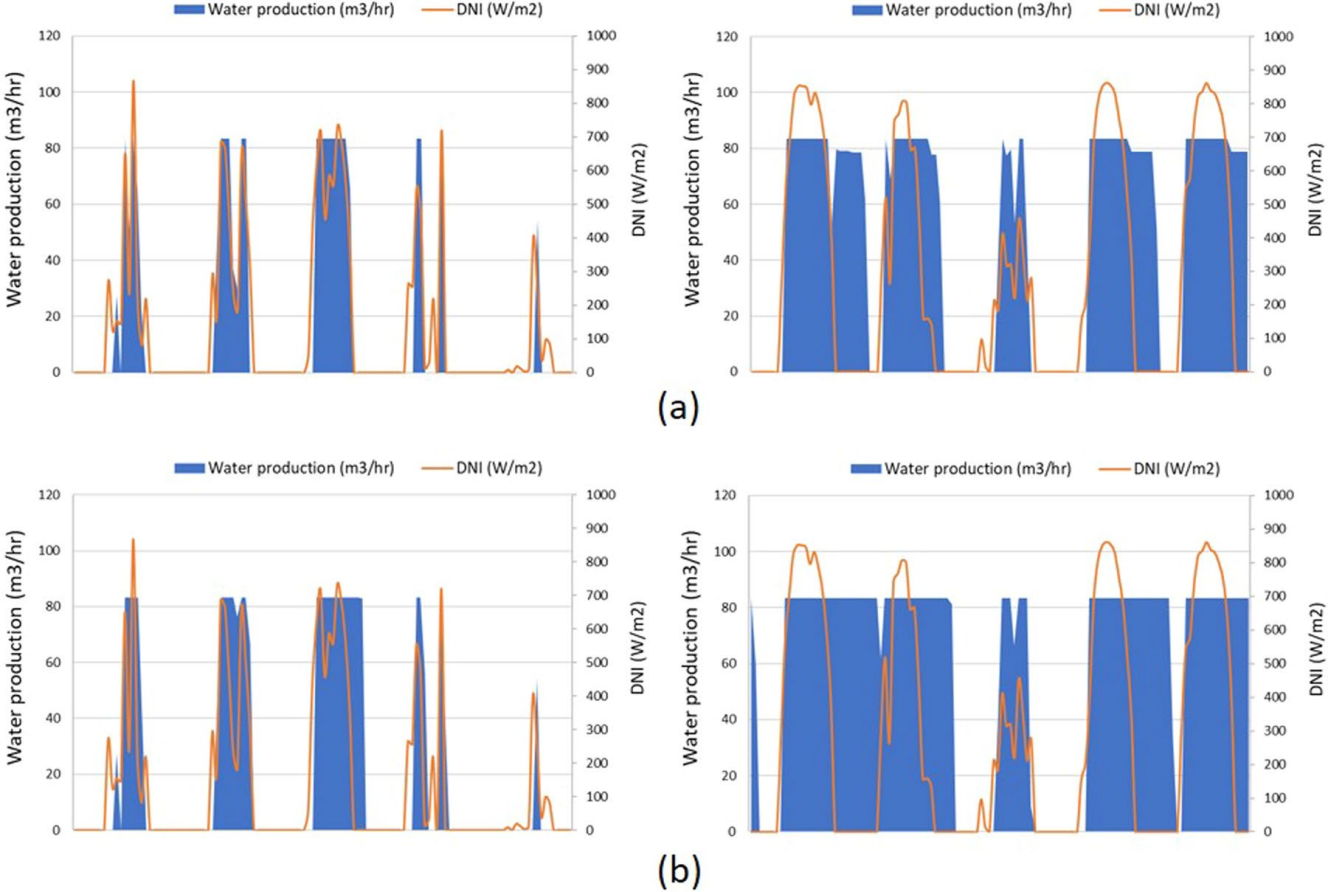
Water production during March 22–26 (left), and during August 5–9 (right) in Almeria, Spain from a 2.5MW_e_ PT-CSP plant coupled with a 2000 m^3^/day MED plant. (**a**) Without thermal energy storage (TES); (**b**) with the addition of a 4-hr TES.

Simulations of an autonomous PT-CSP power generation and a 12-effect LT-MED desalination system operating in Almeria, Spain with a feedwater salinity of 60 g/L, show that during most of the winter, the steam temperature was too low to drive the MED system. On the other hand, in summer, when the temperature of the waste steam reaches 74 °C, the MED system operates at full capacity and the excess heat is curtailed. In such cases, *sedat* suggests the option of adding thermal storage. Figure 11 shows a 5-day performance profile for the CSP/LT-MED plant in March and August, without and with thermal storage.

The average DNI is similar in these two periods (~800 W/m^2^/day), yet a higher temperature in August enables a significant increase in water production. It is also shown by the wider water production daily peaks that a thermal energy storage (TES) system can extend the operation of the MED system by 2 to 3 hours a day in the summer.

Integration with thermal storage system (TES) is also effective in reducing the energy curtailment. In this case, the curtailment is 21.1% in the stand-alone system while only 1.1% waste energy is curtailed after involving the TES.

## Discussion

The software allows the user to interact between the model inputs and simulation results as shown by the bi-directional arrows between the *sedat* block and the outputs cluster in Fig. 2. In this section we discuss a sample of interactions available to the user.

Consider a 2.5 MW_e_ Linear Fresnel Direct Steam Concentrated Solar Power (LFDS-CSP) plant coupled with a 1000 m^3^/day MD-batch desalination plant at Tucson, AZ. The simulation of this system showed that 57% of the LF-DSG plant output was unutilized (curtailed), indicating that the plant was oversized. *Sedat* incorporates performance-based guidance and it issues a warning in the Results Report section when more than 20% energy is unutilized.

**Fig. 12:**
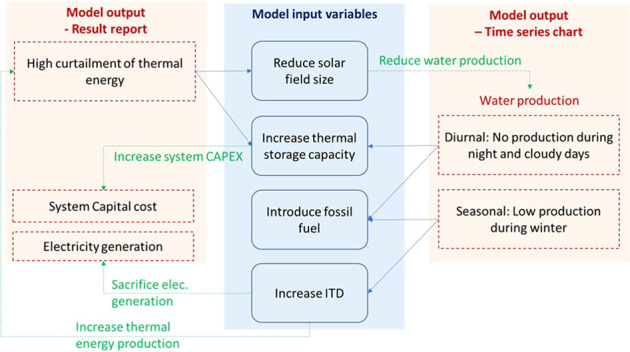
Example of the interactions between model inputs and outputs; suggested options when the model generates high thermal energy curtailment or low water production.

In this example, *sedat* suggested two options (shown in Fig. 12). The first was to reduce the size of the CSP plant, and the second was to store the excess energy and use it during the night.

By reducing the CSP plant capacity to 1 MW_e_, the curtailment dropped to 13.8% but the LCOW reduction was small ($5.15/m^3^ from $5.32/m^3^). However, by adding a 12-hr thermal storage system to the plant, the energy curtailment was reduced to 10.1% while the LCOW was reduced to $4.51 /m^3^. As shown in Table 2, a combination of the two actions produced even more benefit; with a 1-MW_e_ solar field and 12-hr storage system, the curtailment was reduced to 2.3% and the LCOW was reduced further to $4.14/m^3^.

**Table 2:** Example of *sedat* options for reducing energy curtailment and LCOW.

Scenario	Desalination capacity (m3/day)	Solar field size (MW-electricity)	Thermal storage system (hours)	Thermal energy curtailment (%)	LCOW ($/m3)
Base scenario	1000	2.5	0	57.3	5.32
Reduce solar field size	1000	1	0	13.8	5.15
Add thermal storage	1000	2.5	12	10.1	4.51
Thermal storage + reduced solar size	1000	2	12	2.3	4.14

In addition to the Results Report previously shown in Fig. 8, the time-series charts of energy and water production (see Fig. 9) offer data and guidance for further improving the desalination plant performance and LCOW. For example, a user may observe that there is little water production during winter as low ambient temperatures result in condenser waste heat with temperature lower than the required minimum input to thermal desalination technologies. To improve this situation, *sedat* offers two options (as shown in Fig. 10): The first option is to increase the waste heat temperature by increasing the Initial Temperature Difference (ITD). However, this option leads to a decrease of electricity generation. A second option is to add external thermal sources to compensate for low solar energy hours. As shown in Table 3, a combination of the two options gives the greater LCOW reduction.

**Table 3:** Thermal energy generation and LCOW of options available when solar thermal resource is lacking.

Scenario	ITD temperature (°C)	Power cycle efficiency	External heat resources (@ $0.04 /kWh_th_)	Annual thermal energy generation (GWh)	LCOW* ($/m^3^)
Base scenario	40	0.37	No	9.85	4.14
Increased ITD	60	0.30	No	14.77	4.07
Increased ITD + external heat	60	0.30	Yes	14.77	3.52

*An LCOE of 0.05 $/kWh_e_ was assumed in the estimation of LCOW.

## Methods

This section presents the details of the following four steps in developing *sedat*.

1. Compilation of geospatial resource databases and displaying them on map layers.
2. Development of software modules based on open-source libraries to assemble, extract, transform and pass the geospatial data to techno-economic solar generation and desalination components.
3. Development of Graphical User Interface (GUI) components for selecting techno-economic solar and desalination models and required model inputs for analysis. The user interface shows reasonable default values for model parameters. It also provides the ability to substitute local data sources in place of the default data sources.
4. Development of software modules to display desalination model outputs and locations where the estimated cost of water from desalination is lower than that of local municipal and industrial water tariffs. Data can be exported in tables (comma-separated values (CSV) format that can be opened in Excel, R, and other packages).

### Geospatial database compilation and display

Spatial data are inherently important to select an appropriate site for development. This section gives an overview of data collected and the intended use of each one in the project.

#### Solar resource data

Solar input data and weather information are necessary for generating the energy inputs into a solar desalination facility. Models for energy production from solar resources require hourly data on solar irradiation and other meteorological conditions. We integrated data of Direct Normal Irradiance (DNI) and Global Horizontal Irradiance (GHI) for visualization and query in the site selection stage and Typical Meteorological Year (TMY) weather files in the simulations stage. *Sedat* embeds 1,397 TMY weather files from locations around the world. The US data (1,016 locations) were directly extracted from the NSRDB (National Solar Radiation Database)^10^. The TMY files for the locations outside the US were derived from PV-GIS^11^ dataset using PV-GIS API (Application Programming Interface) and were modified applying the NSRDB TMY format. Furthermore, since the PVGIS dataset uses UTC (Coordinated Universal Time) for all locations, the time series for each location were adjusted to the local time according to its time zone.

#### Water resource data

**Fig. 13:**
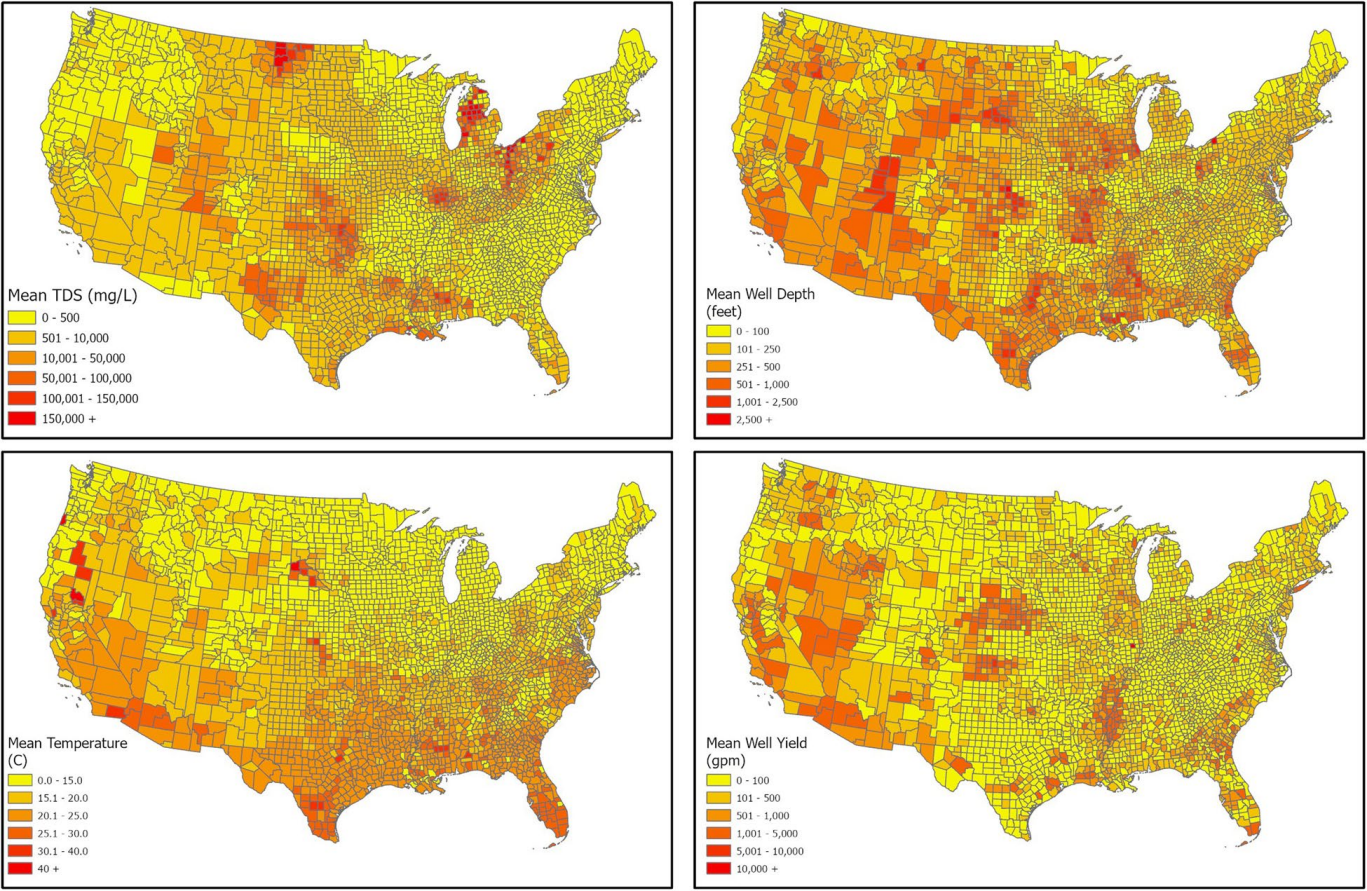
Brackish water resources; (**a**) TDS levels; (**b**) well depth levels; (**c**) water temperatures at well depths; (**d**) mean well yields (data source USGS).

Most of the current US desalination infrastructure utilizes brackish water although seawater desalination is more common world-wide^12^. The prime data source for brackish groundwater is a USGS National Brackish Groundwater Assessment which provides data on occurrence and characterization of brackish groundwater resources^13^. The USGS comprises two datasets: “Dissolved Solids” and “Major Ions”. As part of the aggregation process, parametric statistics (minimum, mean, maximum, variance, standard deviation) were calculated for the total dissolved solids, and depth attributes, water temperatures and well yields (Fig. 13). The data from the brackish water wells were aggregated to county boundaries using the ArcGIS spatial join function. Also, all available data on water temperature were mapped. The aggregation to county level was found useful for statewide visualization of the brackish water characteristics.

**Fig. 14:**
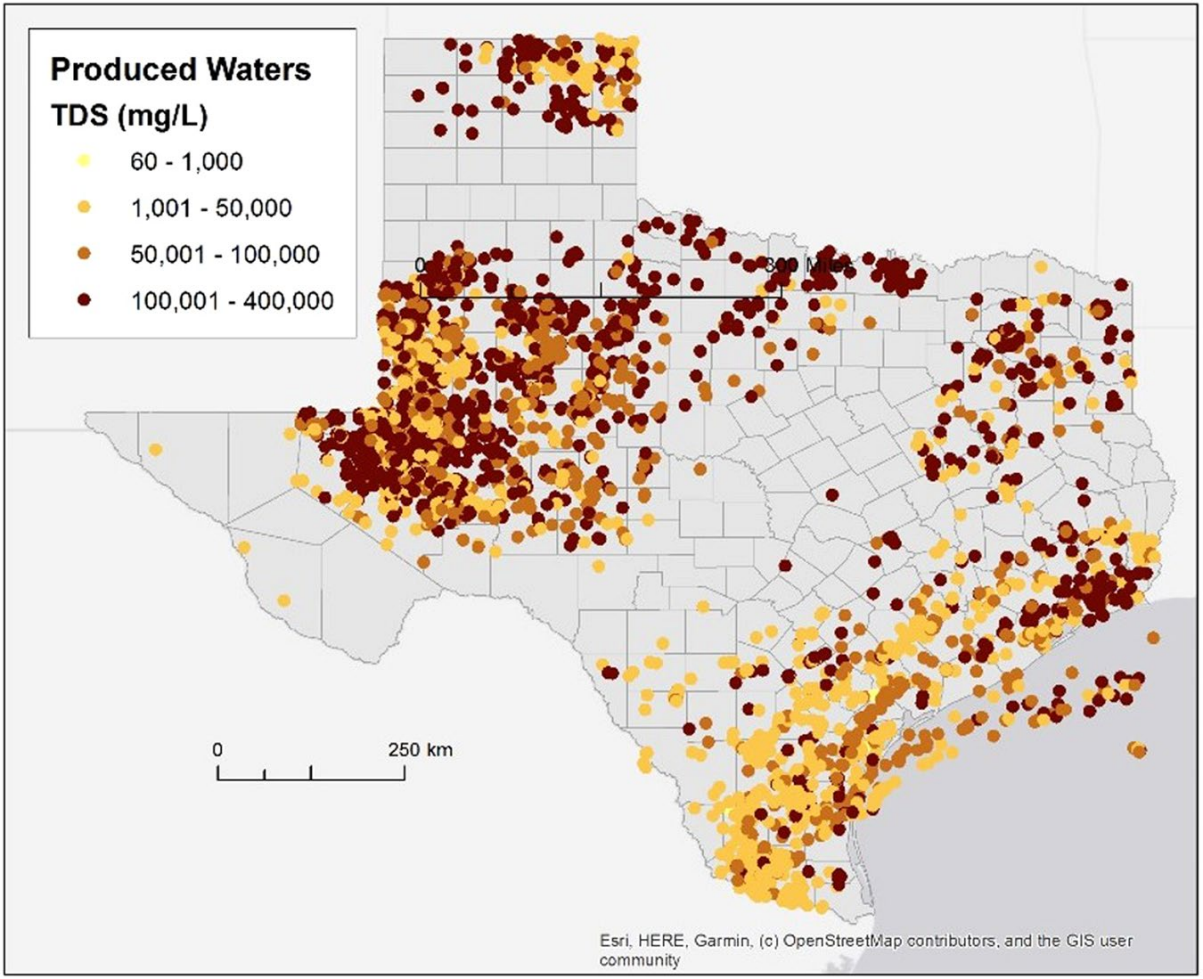
Produced water database extract for Texas (includes offshore wells). (Data source: USGS Produced Waters^13^).

In addition to brackish water, we mapped the availability of other alternative water sources, such as agricultural drainage water, and produced water from oil and gas (O&G) which is more prevalent in Texas; a detail of such O&G produced water resources^14^ is shown in Fig. 14. These alternative water resources are also represented by location and salinity. The feedwater salinity level, as well as the allowed brine concentration determine the appropriate desalination technology and how it impacts the cost of operating these plants^15^. Input water temperature impacts the energy requirements of the system and thus the cost of producing fresh water.

**Fig. 15:**
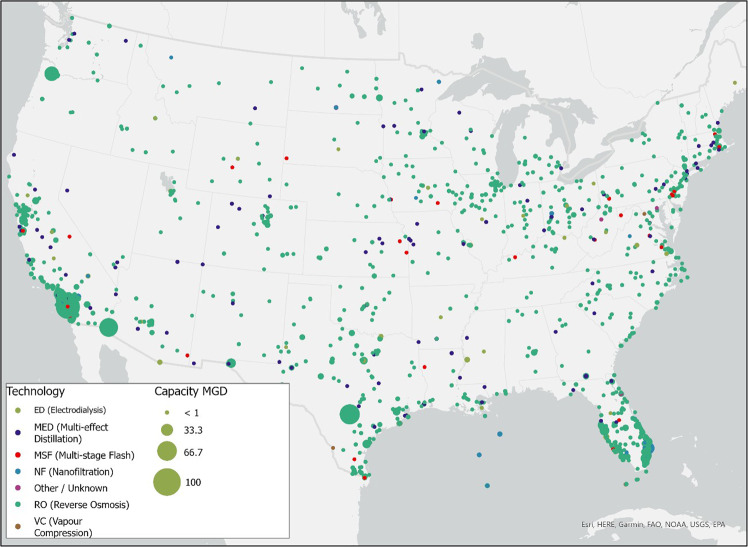
Desalination Plants per desalination technology and plant capacity (GWI 2016 data^17^).

Also, we included the geospatial distribution of U.S. desalination plants as of 2016, courtesy of the Global Water Intelligence (GWI)^16,17^ and tabulated the brine management options available to them. The Texas plants included in *sedat* are shown in Fig. 15.

#### Power plant data

We added the latest US database of power plant locations and capacities available from the Energy Information Agency (EIA) so that the user can examine the possibility of co-locating a desalination plant with a power plant, thereby sharing real estate and grid connectivity. There is also potential for solar thermal desalination to use waste heat from power plants to compliment solar resources. Gingerich and Mauter^18^ estimated that the US generated 14.6 billion GJ of electricity and 26.4 billion GJ of waste heat via coal, nuclear, and natural gas power plants. Of this waste heat, 99% is condenser heat discharged to the environment at or below 41.5 °C which under most conditions is too low to be practically recoverable. The remaining 1% of waste heat is discharged in exhaust streams at temperatures between 91 and 543 °C which if recovered could be used in thermal desalination plants that are near the power plants. *Sedat* guides the user by showing straight-line distances between a selected site and near-by power plants.

#### Water transfer and distribution infrastructure

Existing conveyance water networks can serve the delivery of desalinated water from the plant to customers; thus, the distance of the selected site to the nearest network is an important consideration given the cost of building a new water delivery system. Existing water networks are represented in *sedat* by two data sources: existing canals and aqueducts^19^ and a proxy for local municipal water systems based on road locations^20^. Canals and aqueducts are represented visually in the system, and the straight-line distance from a selected site is calculated and shown on the map when a user selects a location. The water network proxy locations are stored, but not displayed in the system. The basemap in the application includes a road overlay that is separate from the water network. The road network has been subset to include types that are more likely to be residential; interstates and other road types that are mostly rural have been excluded. The network along a generalized road network are presumed to likely have water transportation networks. The distance to the nearest point is calculated based on the road network proxy.

#### Energy and water markets

Electricity rates and fuel prices from the OpenEI Application Programming Interface (API) are being used for cost comparisons with solar-powered desalination. A script was developed to construct a link to the web site; it references the latest available data on the web site, which is updated regularly.

Water price data were downloaded from the free, open-access IBNET water tariff database^21^, a joint product of the Global Water Intelligence (GWI) and the International Benchmarking Network of the World Bank (IBNET). It includes data on water utilities for 151 national jurisdictions, for a range of recent years up to 2017 (year range varies greatly by country and utility) on service and utility parameters (Benchmark Database), and water tariffs for 211 jurisdictions (Tariffs database). Information includes cost recovery, population served, financial performance, non-revenue water, residential and total supply, and total production. Data can be called up by utility, by group of utilities, and by comparison between utilities, enabling both country and global level comparison for individual utilities.

The data are multi-faceted with multiple price entries associated with fixed and consumption charges for various scales of consumption. To reduce complexity and maintain the user-friendly format of *sedat*, only the consumption charges and the corresponding consumption levels are shown. In addition, links to local utility web-sites were added on the maps, where the user can see the pricing detail and get price updates in the future.

#### Regulatory data

A preliminary permitting requirement database was compiled with examples of regulatory and permitting requirements from Texas^22^, Arizona^23^, Nevada^24^, Florida^25^, California^26^ and Colorado^27^. The development of this database followed the following steps: a) Searching the websites of state and/or county water management and environmental quality agencies, b) researching, collating and studying project development reports for existing desalination plants with a specific focus on permitting experience and challenges. Information on the permitting requirements applicable to existing desalination plants was compiled, and the associated state and county permitting requirements where synthesized into tabular forms that are shown to the user of *sedat*, together with links to associated agencies and permitting forms per state and county as available and applicable. Where available, the costs of the permitting process were included. The data we were able to gather do not present a complete regulatory picture, but they are intended to show elements of the types of permits required and associated agencies, setting a foundation for further examination.

#### Population growth data

Population growth estimates for U.S. counties consistent with the Shared Socioeconomic Pathways (SSP)^28,29^ are integrated in the application to show areas of projected population growth or decline under each pathway scenarios. In the GUI, the layer is available and rendered based on growth or decline; when a user selects a county, the population growth curves are shown for all five SSP scenarios along with the mean growth curve for all of the scenarios. In this way, users can see if a proposed site is within or near a county that is expected to have a growing demand for water.

### Software modules for data selection, mapping and integration with the techno-economic models

Software modules using open-source libraries have been prototyped to select the appropriate data based on the user’s selected site.

Spatial queries (nearest features, distances, overlapping features) are implemented in Python with the use of OSGeo and other open libraries, including Fiona, Shapely, Xarray, and SciPy.spatial. As shown in Fig. 3, once a user selects a location on the map, details of the location chosen are shown or made available via hyperlinks, including the DNI and GHI values, utility prices (fuel and electricity), and regulatory information at the state and local levels. Distances and details on the closest desalination facility, water network proxy, canal or aqueduct, and power plants are assessed and lines to each of these closest locations are drawn on the map.

Adequate performance of the site selection portion of the GUI (site selection query completing in less than a minute) required several strategies. Fiona, Shapely and SciPy.spatial were used to pre-process the GIS data and implement a rapid lookup of the nearest feature to a user-selected site through the creation of spatial indexes, thus KDTrees for Points and RTrees for Polygons. Note that line features were converted to points (one per vertex in the linear feature) and queried based on the KDTree of the points to find the nearest feature. The line features were also generalized using a tolerance of 15 m, significantly reducing the level of detail in the data while not impacting the location precision significantly.

The points and polygons are queried in a two-step process: the index for each polygon spatial layer is queried to quickly identify candidates for a match; if only one candidate is found it is read directly from the GIS layer, greatly reducing the memory footprint required to read in the data. The indexes (bounding boxes) for polygons can overlap; if multiple candidates are found, the candidates are read into memory (again, a much smaller footprint than the entire dataset) and the user-selected point is compared to each candidate using the “contains” operation to find the matching polygon. For point locations, the KDTree is queried to find the closest point, which is read into memory from the selected dataset. For large point layers (e.g., canals and aqueducts, and water proxy locations) a two-stage query is employed. The state and county that a user-selected point falls within is first determined by querying as described above for all polygons. The state and county code are used to identify the index file for each road and canal location and these are stored separately to reduce memory size. The road network (water proxy) includes 81,185,710 point locations within the United States and, if read in at run-time for location lookup, it results in poor performance. Our initial approach divided the water network proxy by State, but this was still slow (more than 60 seconds for a road query in a large state such as California). We further subdivided the water network proxy into county-level files, which improved performance by a factor of two in large States. In addition to speed of access, the large data volume is also an issue. If uncompressed, the shapefiles (and associated KDTree index files) require 46.1 GB on disk; the compressed shapefiles and associated KDTRee index files only require 4.6 GB on disk. Testing of the opening speed for compressed shapefiles using the Python library, Fiona, versus uncompressed shapefiles resulted in a response time difference of approximately 0.04 seconds for the largest zip archive (14.7 MB).

Wrapper modules for the techno-economic models in Python have been developed. These modules have the input parameters required for the models, and will be called by the code supporting the user interface (passing the input parameters collected from the GIS data and user). This is the integration point between the GUI and the modeling components of the software application.

### GUI model development and integration

#### Solar energy generation models from SAM

Concentrated solar power (CSP) and photovoltaics (PV) models, including the associated financial models, from NREL’s System Advisor Model (SAM) were tested for integration into *sedat*. SAM provides annual-hourly simulation for different plant parameters depending on hourly weather conditions. However, since these models were designed for electricity rather than heat production, the source code was modified to obtain variables needed for integration with solar thermal desalination models (e.g., condenser temperature and exhaust steam mass flow rate in the power block). The SAM models were integrated into sedat using Python wrappers. However, in order to develop a graphical user interface (GUI) for *sedat*, the wrappers created from SAM for CSP modules and financial modules needed substantial modifications and restructuring. The Python wrappers for these models contain more than 300 input variables for each module which were being initialized in the wrapper, and “methods” were created in each wrapper corresponding to sub-sections on the SAM user interface; “methods” are a type of function in Python classes. However, the presence of more than 300 variables for each SAM model made this approach tedious. Further, programmatically calling multiple methods for each model’s element was not the most efficient way of integration. Subsequently, an interface was developed using the DataTable function in DashPlotly to construct the frame of input variables and JavaScript Object Notation (JSON) to structure all the variables of each model and adapt it to our GUI.

JSON captures the structure of the input fields on the user interface along with assigning values and reading properties for each variable. JSON objects can transmit attribute-value pairs, arrays and any other serializable data objects; these were used to store and communicate data used in modeling solar and desalination plants of any type. This approach consisted of creating Python wrappers for each model from the user interface of SAM. Each variable value from the wrapper was matched with its defaulted value from SAM and added to a JSON file.

The functionality and simulations run by SAM are imported into the software by using the Python scripts generated by creating a SAM wrapper. The wrapper initializes all the variables used for a model within SAM, compiles the source code of SAM and executes the models.

The new wrappers were further modified to reflect software engineering best practices. Unit tests were added to each wrapper and a logger was set up for each application which can be accessed from any module in the code. These functionalities allow the programmers working on the development to debug the code and improve the efficiency of integrating different modules. Logic checks were added to handle rules governing input parameters, such as when the change of an option on one tab can impact the default values on a different tab. This logic was also added to the callback functions. Also, to guide the validity of user input choices, “Constraints”, and “Connections” were implemented. The “Constraints” attribute establishes the range of valid inputs in each technical solar and desalination model parameter. “Connections” describe relationships between certain pairs of inputs.

The JSON file is used to build the GUI interface by iterating through each tab, section, and subsection in the SAM user interface and creating the corresponding menu elements in the Dash framework. This code can be reused to create menu structures that represent each of the SAM models without having to manually program the menu elements; each model can have a menu system automatically generated from the JSON file. Logic for loading the various models was created in the Python code so that the appropriate menu system is loaded based on the user-selected model.

Our approach involved capturing the structure of the variables on the GUI inside the JSON in a non-repetitive manner along with matching default values of all the SAM variables from a sub-branch of the SAM GitHub repository^30^ that includes JSON files with all of the model variables and default values. The default values of different model variables in SAM are populated in from these files. We identified these files in SAM GitHub and used them in the development process for our software, saving time and effort over the manual process we had previously used. The JSON file created for input variables captures the structure of the GUI by adding variables as list elements inside dictionaries. The dictionaries contain the name of the tab, section or sub-section on the GUI as a dictionary key and have the variables, sub-section or section elements as dictionary values. In this manner, it is easier to collect different variables belonging to each GUI section while generating the GUI programmatically.

**Fig. 16:**
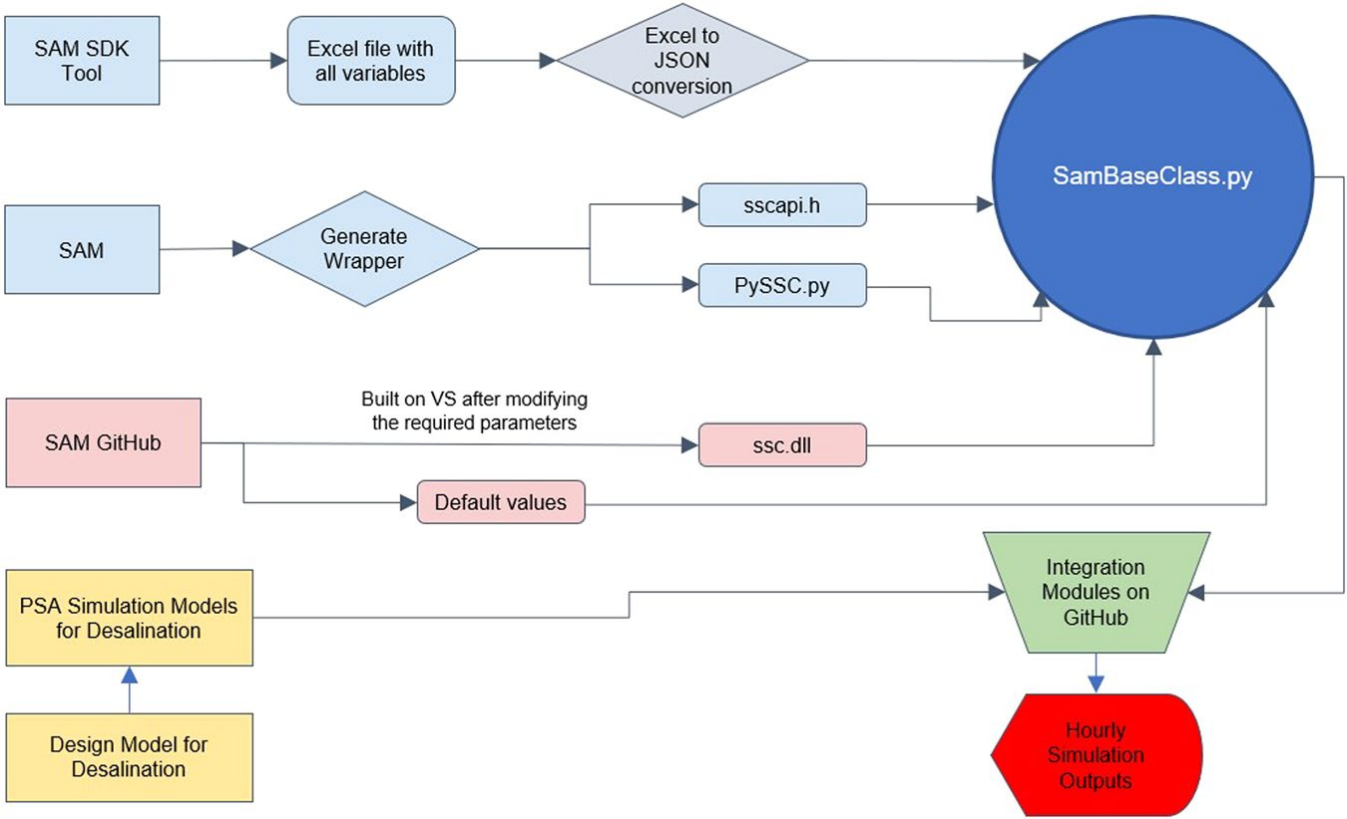
Schematic of the elements and files used in the solar and desalination model integration.

The different files associated in this integration are structured in folders as shown in Fig. 16. SamBaseClass.py is the Python file that integrates and uses these files. The files generated from the wrapper (PySSC.py, sscapi.h, ssc.dll for Windows, and ssc.dylib for MacOS) are also placed inside the same folder. The compilation is a one-time process; once created, the files are included in *sedat* and used for all desalination models that require solar energy inputs. New versions of SAM, along with new desalination or solar thermal technologies, can be incorporated into our software by just modifying the files mentioned earlier.

Each CSP or PV model can be used as a stand-alone model, or combined with one of the six financial models. All these models are integrated in a JSON structure and tested to ensure that nothing was missed in the translation. For verifying the implementation of the SAM models in our software, a number of inputs were varied to compare the outputs of the model at its source with the JSON platform.

However, some parameter dependencies were described in the SAM user interface, rather than in the source code and those had to be restructured in the *sedat* GUI. To implement such dependencies, we identified the equations embedded in the SAM interface from the SAM installation folder. Then we isolated the ones that calculate input variables. For example, in SAM’s Molten Salt Power Tower model, the number of loops is calculated by:

$$nLoops=\frac{specified\_solar\_multiple\times sm1\_aperture}{loop\_aperture}$$

*nLoops*: Actual number of loops

*specified_solar_multiple*: User defined solar multiple (SM)

*sm1_aperture*: Total required aperture, SM = 1 (m^2^)

*loop_aperture*: Single loop aperture (m^2^)

On the right-hand side of the equation, “specified_solar_multiple” is one of the model input variables, and the other two are intermediate variables that are calculated in the GUI but not used in model simulation. The total required aperture “sm1_aperture” is a function of design capacity, design point DNI and optical parameters. The single loop aperture “loop_aperture” is a function of collector area and number of modules.

Thus, the number of loops is dependent on multiple other input variables and it is necessary to implement such relationships to fit the cases. The implementation work flow mainly includes building the structure (e.g., callback functions) to dynamically link those dependent variables, updating the target variable in the GUI in real time and visualizing them. In summary, the procedure of implementing the SAM models into *sedat* includes the following steps:

1. Identify the valid input/output variables of a SAM model and organize them into a JSON file, which can be used for a Dash data table in the GUI.

2. Create a JSON file containing the default values for the input variables.

3. Identify all the parameter dependencies from SAM GUI and create callback functions accordingly.

4. Connect the location of the weather file to the map selection information.

5. Connect the SAM model results to the desalination and cost model.

6. Create warnings or suggestions to users when certain options are chosen.

#### Desalination models

*Sedat* incorporates several well-tested desalination models; a list is shown in Table 4.

**Table 4:** Summary description of desalination models included in *sedat*.

Desalination technologies	Model development
MED and MD	These models were developed and validated at pilot-scale at PSA^5–9^. Some models were written in MATLAB and other were based on Engineering Equations Solver (EES). The EES-based models were approximated with empirical equations coded in Python and the MATLAB-based MED design models were converted to simulation ones also in Python.
Forward Osmosis (FO)	The FO model was developed and validated by Trevi Co.^45^; it was translated into Python and generalized for wide input ranges at Columbia University.
Multi-pass Reverse Osmosis (RO), and Osmotically Assisted Reverse Osmosis (OARO)	Developed in Matlab and Python at Columbia University^15,46–48^. The multi-pass RO model was designed for high purity permeate production for electrolyzer and other industrial applications, whereas the OARO model is used in minimal and zero-liquid-discharge pathways, for comparison with thermal desalination technologies.
Hybrids (RO-FO and RO-batch-VAGMD)	Developed at Columbia University. Designed for low cost and/or minimum liquid discharge applications.

The inputs and outputs for desalination models were constructed in the same format as SAM variables, in order to be integrated into the same GUI frame. However, unlike SAM models which are based on C++ source code and require the connection between the inputs and models to be built, desalination models are constructed in a way that can directly import the inputs from the GUI and execute.

**Fig. 17:**
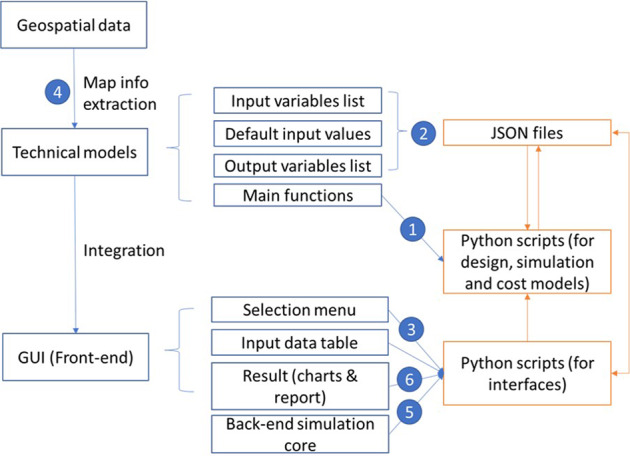
Outline of the *sedat* modular architecture.

The modular architecture of *sedat* allows the addition of new solar and desalination models, in the six steps shown in Fig. 17 and summarized below:

1. Develop Python scripts for the models. For solar models, the output should include the hourly energy generation, LCOH and LCOE. For desalination, design, simulation and cost models should be developed
2. Create the JSON files that describe the input variables and their default values
3. Configure the possible combinations of the new models so that it can be selected from the menu
4. Connect the input variables from the map to the models (e.g., the weather file of a selected location and the feedwater salinity)
5. Connect the input and output of the design, simulation and financial models
6. Configure the output variables for the result chart and report

### Graphical user interface (gui) structure

**Fig. 18:**
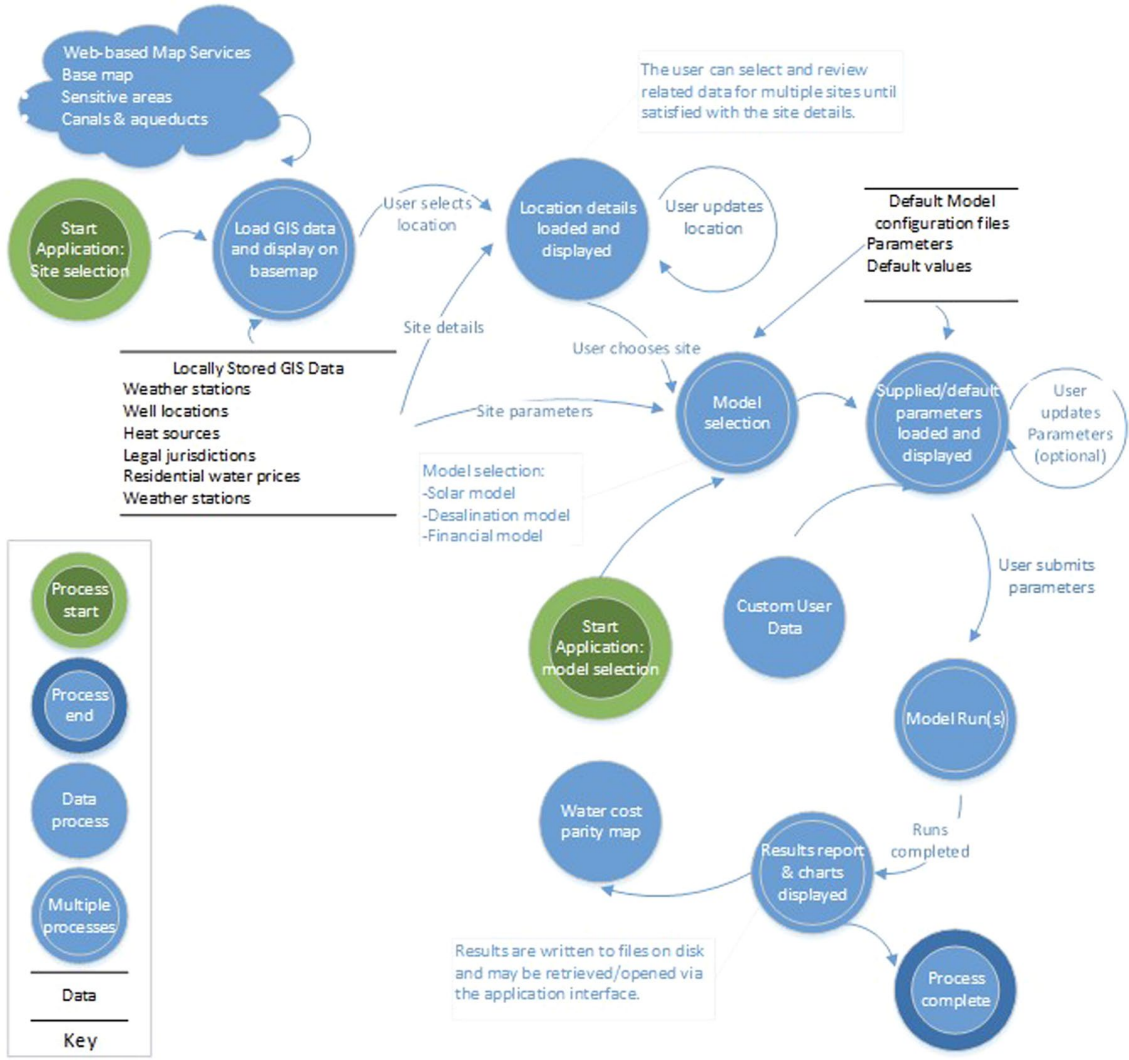
Schematic of the data flow and model runs in *sedat*.

The data flow diagram shown in Fig. 18 walks through a high-level workflow for site and model selection using the desktop application for a site-selection driven workflow.

In a typical workflow, an user would work through the steps shown in the data workflow diagram to complete an analysis: site selection, model selection, review of input parameters, model run, review results. The site selection stage can be skipped, in which case the model software will use the last site selected by the user for the model run. In model selection, the user selects a supported combination of solar thermal model, desalination model, and financial model. The site selection and parameter review workflows can be iterative: in site selection, multiple sites can be reviewed before proceeding to model selection; in parameter review multiple parameters can be edited before running the models. During the parameter review, a user can replace the default input parameters, generally through direct editing in the GUI. If the user has a custom weather file in the documented TMY format, it can be used as a model input by entering the full path to the file. Additionally, parameters generated from SAM software can be used as an input by uploading a SAM generated JSON file. Once the models are executed, the results are presented to the user with hourly, daily and weekly time-series solar generation and desalination plant performance (e.g., condenser steam temperature, steam flow rate, waste heat generation, water production, fossil fuel use, and storage status). A link to the report page will load a summary of input model parameters and outputs to be displayed, along with links to download the model outputs. An additional link for the results map will display locations where water prices from utilities are equal to or greater than the LCOW calculated by the model. The results can be refined by adjusting the factor for filtering; i.e., the water parity can be dynamically adjusted to show utilities that are less or more expensive than the calculated LCOW. At this point the workflow ends. The process could be started again, or the application closed.

Existing sources can be replaced with more recent versions for spatial query and new data can be added by adding layers in the map file. The software can be expanded to include synergistic renewable energy sources that could be used to meet the energy demand for any proposed water treatment infrastructure. One example is considering solar thermal, geothermal, and waste heat resources to collectively drive thermal desalination plants. Another example is to assess the solar and wind complementarity at high temporal resolution to examine the extent that VRE alone can power desalination plants.

## Data Availability

All the open-source data inputs of *sedat* are accessible through figshare^31^ (10.6084/m9.figshare.c.5874125.v5). Viewing or using these data requires GIS software, such as Esri ArcGIS or the open source QGIS application.

## Code availability

All source code for *sedat* is made available through github under the academic free license, at the time of writing. The code is hosted at https://github.com/gyetman/DOE_CSP_PROJECT.
